# An unusual case of crescentic lupus nephritis presenting with normal renal function

**DOI:** 10.4155/fso.15.4

**Published:** 2015-11-01

**Authors:** Sandhya Manohar, Chamundeswari Subramanian, Kameswari Lakshmi

**Affiliations:** 1 Department of Medicine, Montefiore New Rochelle Hospital, 16 Guion Pl, New Rochelle, NY 10802, USA; 2 Division of Nephrology, Department of Medicine, Montefiore New Rochelle Hospital, 16 Guion Pl, New Rochelle, NY 10802, USA

**Keywords:** crescents, lupus nephritis, normal renal function

## Abstract

Lupus nephritis is a life-threatening manifestation of systemic lupus erythematosus (SLE). This is commonly suspected when lupus patients present with elevated serum creatinine levels. But it is important to be aware that even patients with advanced disease in the kidney from SLE can have normal renal function, thus requiring a high index of suspicion. We present the case of a patient who presented with nonspecific musculoskeletal symptoms and was diagnosed with SLE. He also had nephrotic range proteinuria but his serum creatinine was normal. A renal biopsy revealed diffuse proliferative crescentic lupus nephritis. We have reviewed the literature for correlation between crescents; a sign of severe glomerular damage and creatinine levels.

## Abstract (lay abstract)

Lupus nephritis is a life threatening disease. Diagnosing it can be a challenge. It is suspected when patients with lupus show abnormal kidney function. A definite diagnosis is made from a kidney biopsy but the decision on the timing of the biopsy is a difficult one to make. We present the case of a young man who presented with body aches and a workup led to a diagnosis of lupus. His urine was suspicious but his kidney function was normal. A decision was made to proceed with the biopsy and rightly so, as it revealed advanced lupus nephritis.

**Figure 1.**
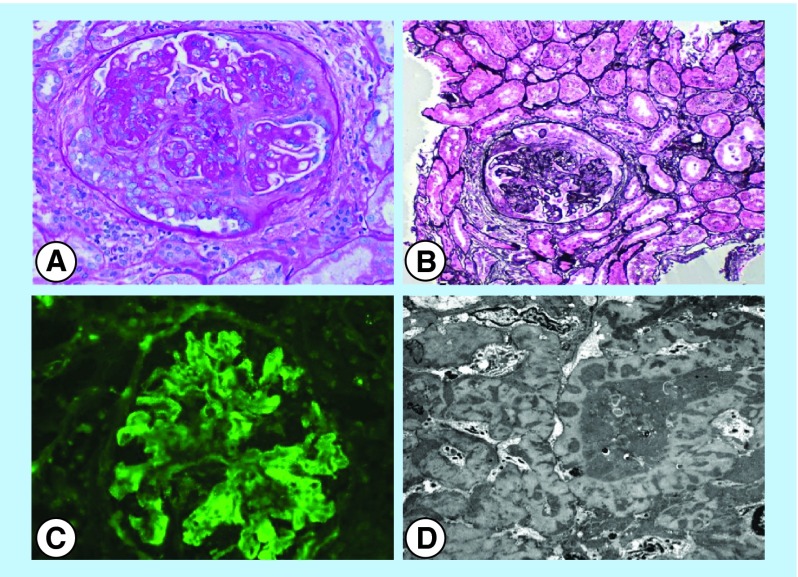
**Renal biopsy images.** **(A&B)** Light microscopy of sections of renal cortex and medulla with active cellular and fibrocellular crescents. Frequent spike formation and segmental subendothelial deposits are seen. **(C)** Direct immunofluorescence of the renal cortex shows glomeruli with granular staining in the mesangial and capillary walls for IgG, IgA, IgM, C3, C1q, kappa and lambda light chains. **(D)** Electron microscopy of a glomerulus exhibiting immune-type electron-dense deposits in the subepithelial, intramembranous and mesangial locations. Glomerular basement membranes are irregularly thickened and distorted by the deposits.

Involvement of the kidney in systemic lupus erythematosus (SLE) is a common and leading cause of mortality among these patients [1]. Patients are suspected to have lupus nephritis when routine labs reveal abnormal renal function or suspicious findings are seen on urinalysis, such as proteinuria, hematuria or active sediment containing red cell casts. Based on those findings, the class of nephritis may be predicted but it does not necessarily correlate with the actual histological picture on the renal biopsy [2]. We present an unusual case of a patient who presented with nephrotic range proteinuria and a normal renal function but renal biopsy showed diffuse proliferative crescentic lupus nephritis. A review of the literature for the prevalence and the possible reasons of the normal renal function in this clinical setting follows in the discussion.

## Case report

A 47-year-old Hispanic male presented to our emergency department with severe neck pain. He has had intermittent episodes of neck pain with radiation down his spine and lower extremities for the past year. It was described as a sharp and sometimes cramping, 8–10/10 in intensity, intermittent and was triggered by activity like lifting heavy objects. Two weeks prior to admission he was evaluated at a local clinic for the same complaints, treated with ibuprofen and referred to our rheumatology clinic where a workup was in progress.

His pain became more intense prompting him to seek medical attention and was admitted to our hospital for evaluation. He noted that he had been more fatigued lately with generalized malaise. He had difficulty climbing stairs and moving from a sitting to a standing position but was able to eat with a fork/spoon and comb his hair without any difficulty. He reported that his fingers turned blue on exposure to cold weather. He occasionally had joint pains with swelling and redness of multiple joints, associated with morning stiffness lasting at least 30 min. He also reported noticing frothy urine for the past year.

His past medical history was significant for hypothyroidism and hypertension. His medications consisted of lisinopril, aspirin and levothyroxine. He had no allergies. He denied any family history of thyroid, autoimmune or rheumatological diseases. He was a landscaper by profession. He denied smoking cigarettes, alcohol use or illicit drug use.

On examination, he was a young Hispanic male in some distress from the neck pain. He had unremarkable vital signs with a BMI of 25. His physical examination was normal. X-rays of his chest and spine were normal. His blood work revealed anemia with hemoglobin of 11 g/dl, marked hypoalbuminemia of 1.7 g/dl and a total protein of 3.6 mg/dl. He had a blood urea nitrogen of 33 mg/dl and serum creatinine of 1.0 mg/dl. His triglycerides were elevated at 265 mg/dl, cholesterol of 209 mg/dl and a calculated LDL of 132 mg/dl. His previous blood work done a few days ago at our rheumatology clinic showed an elevated erythrocyte sedimentation rate of 53 mm/h, TSH 10.6 and free T4 1.27 ng/ml. A urinalysis showed 1+ blood with 9 RBC, hyaline and granular casts but no RBC casts, protein of greater than 600 mg/dl with a urine protein-creatinine ratio of 4 grams/gram of creatinine. HIV, hepatitis B and C serologies were negative. Antinuclear antibody was positive with a titer of 1:640 and a peripheral pattern.

A nephrologist and a rheumatologist were consulted. Given normal renal function, microscopic hematuria and massive proteinuria, membranous nephropathy secondary to SLE was suspected. Further workup revealed a low C3 of 76 mg/dl, a normal C4 of 36 mg/dl and the anti-dsDNA titer was positive at 29 IU/ml. Renal biopsy was done and it revealed immune-complex-mediated diffuse segmental proliferative and membranous nephritis with features of focal cellular and fibrocellular crescents (8–10%). The segmental active glomerular lesions were in approximately 79–83% of sampled glomeruli, which were characterized by segmental endocapillary hypercellularity, hyaline ‘pseudothrombi’ (intraluminal immune aggregates), duplication of glomerular basement membrane (GBM) and, cellular and fibrocellular crescent. There was no significant tubulointerstitial chronic injury. The biopsy was diagnostic for lupus nephritis class IV-S (A/C) and V by ISN/RPS 2004 classification (Figure 1). He was started on prednisone, hydroxychloroquine and mycophenolate mofetil. Four months after initiating therapy he started to show improvement in his proteinuria. He continues to have a normal renal function (Table 1).

## Discussion

Lupus is a multisystem organ disease with kidney being one of the most commonly affected organs. Nephritis has been reported to afflict 27–45% of patients with SLE [3]. Approximately 10–30% of these patients progress to end-stage renal disease and despite improvements in overall care, long-term mortality remains high [4]. Involvement of the kidney is suspected when there is a worsening of renal function on routine labs or if the patient is noted to have an abnormal urinalysis. It is interesting to note that contrary to popular belief, only 25–50% of the patients have abnormal renal function [5]. The clinicians need to have a high index of suspicion in order to diagnose the disease early on in the natural history of disease progression. For unless it is identified and treated in a timely manner, patients with proliferative form of lupus nephritis progress to chronic renal failure [6].

Lupus nephritis is classified based on renal histology findings (Table 2) and crescentic glomerulonephritis is not a specific disease but a histologic manifestation of glomerular damage. Crescents are formed when the immune complexes cause glomerular injury by disruption of the basement membrane, thereby allowing circulating leukocytes, coagulation factors and inflammatory mediators to enter the Bowman's space and result in its obliteration [7]. The development of crescents in the kidney, in general, is considered as a sign of severe glomerular injury [8]. A crescent score is calculated in these patients by dividing the number of crescentic glomeruli and the number of nonglobally sclerotic glomeruli, to define the magnitude of glomerular injury. Involvement of less than 50% of the glomeruli is considered segmental while the terms severe and extensive are used for crescents involving more than 50 and 80% of the glomeruli, respectively [8]. In a study by Yu *et al*., it was found that crescentic glomerulonephritis was not rare, it accounted for 10.1% of the total biopsy-proven lupus nephritis and 21.7% of lupus nephritis class IV-G [9]. But Sumethkul *et al*. showed that lupus nephritis with crescentic glomerulonephritis accounted for 51.6% (32/62) of all patients with biopsy-proven crescentic glomerulonephritis [8]. Despite the destructive nature of the crescent formation they have not been attributed to affect the renal function. Zeng Tang *et al*. noted the serum creatinine was >1.4 mg/dl in approximately 76% of their patients with diffuse crescentic glomerulonephritis and the remaining (˜34%) were in the normal range [10].

There have been many theories proposed to explain the normal renal function in patients with crescentic glomerulonephritis. Some have attributed this to a sampling error, resulting in histology that is not representative of the overall picture. There is also evidence to suggest that patients with significant tubular lesions are likely to have an abnormal renal function [12,13]. Our patient had no significant involvement of the tubules and it can be contemplated that this may have a played a role in his relatively preserved renal function. Other factors that have been predicted to affect the renal function are evidence of chronicity like glomerular sclerosis, fibrous crescents (unlike fibrocellular which are more active lesions), tubular atrophy and interstitial fibrosis [13]. The proliferative forms of lupus nephritis tend to have a relatively poorer renal function [14]. Of these, Class III (focal lupus nephritis) and IV (diffuse lupus nephritis) are usually considered to be the more severe forms and are aggressively treated [5]. Long-term retrospective studies on patients with class IV disease have shown that approximately 50% of them had worsening of their renal function over 5–10 year period and a significant number of those had abnormal renal function at the time of diagnosis [15].

## Conclusion & future perspective

Clinicians should have a high index of suspicion for renal involvement in patients with urinary abnormalities alone. Normal renal function does not necessarily rule out renal involvement. Any urinary abnormalities in these patients have to be addressed and managed expeditiously. Timely initiation of appropriate therapy can reduce the likelihood of progression to chronic kidney disease. Contrary to popular belief, even though crescentic glomerulonephritis is the most severe form of renal injury it may not translate into loss of kidney function. Our patient had an atypical presentation of Class IV lupus nephritis with no RBC casts or elevated serum creatinine. Renal biopsy is a valuable tool to guide therapy and should strongly be considered in patients with suspected kidney disease with SLE.

**Table 1.** **Trend of laboratory data.**

**Variable**	**Reference range**	**Prior to admission**	**Day of admission**	**1 month follow-up**	**7 month follow-up**
Serum creatinine (mg/dl)	0.5–1.2	0.93	1.00	0.8	0.75
Protein/creatinine ratio (g/g of creatinine)	0–0.2	4.0	4.0	9.1	3.6
Anti-dsDNA antibody (IU/ml)	<4	–	29	2	<1
Complement C3 (mg/dl)	90–180	–	48	59	105
Complement C4 (mg/dl)	16–47	–	23	28	36
Erythrocyte sedimentation rate (mm/h)	0–15	53	49	13	3
Albumin (g/dl)	3.2–5.5	1.7	1.5	1.5	3.0

**Table 2.** **The 2003 International Society of Nephrology/Renal Pathology Society classification of lupus nephritis.**

Class I	Minimal mesangial lupus nephritis
Class II	Mesangial proliferative lupus nephritis
Class III	Focal lupus nephritis
Class IV	Diffuse segmental (IV-S) or global (IV-G) lupus nephritis
Class V	Membranous lupus nephritis
Class VI	Advanced sclerosing lupus nephritis

Reproduced with permission from [11] © Nature Publishing Group (2004).

**Executive summary**

Renal involvement in lupus is considered a life-threatening manifestation and a high index of suspicion is needed in making a diagnosis of lupus nephritis.
Any urinary abnormality in lupus patients, despite a normal renal function, should be managed with a sense of urgency.
Patients with abnormal renal function in lupus nephritis are likely to have significant tubular involvement and evidence of chronicity on their kidney biopsy.
The presence of crescents, though a sign of severe glomerular damage, may not reflect in the patient's glomerular filtration rate.

## References

[1] Bernatsky S, Boivin JF, Joseph L (2006). Mortality in systemic lupus erythematosus. *Arthritis Rheum.*.

[2] Zweiman B, Kornblum J, Cornog J, Hildreth EA (1968). The prognosis of lupus nephritis: role of clinical-pathologic correlation. *Ann. Intern. Med.*.

[3] Medpage Today Death rates still high in Lupus Nephritis. http://www.medpagetoday.com/Rheumatology/Lupus/20453.

[4] Appel GB, Cohen DJ, Pirani CL, Meltzer JI, Estes D (1987). Long-term follow-up of patients with lupus nephritis. A study based on the classification of the World Health Organization. *Am. J. Med.*.

[5] Cameron JS (1999). Lupus Nephritis. *J. Am. Soc. Nephrol.*.

[6] Contreras G, Pardo V, Cely C (2005). Factors associated with poor outcomes in patients with lupus nephritis. *Lupus*.

[7] Tipping PG, Kitching AR, Cunningham MA, Holdsworth SR (1999). Immunopathogenesis of crescentic glomerulonephritis. *Curr. Opin. Nephrol. Hypertens.*.

[8] Sumethkul V, Chalermsanyakorn P, Changsirikulchai S, Radinahamed P (2000). Lupus nephritis: a challenging cause of rapidly progressive crescentic glomerulonephritis. *Lupus*.

[9] Yu F, Tan Y, Liu G, Wang S, Zou W, Zhao M (2009). Clinicopathological characteristics and outcomes of patients with crescentic lupus nephritis. *Kidney Int.*.

[10] Tang Z, Wang Z, Zhang H (2009). Clinical features and renal outcome in lupus patients with diffuse crescentic glomerulonephritis. *Rheumatol. Int.*.

[11] Weening JJ, D'Agati VD, Schwartz MM (2004). The classification of glomerulonephritis in systemic lupus erythematosus revisited. *J. Am. Soc. Nephrol.*.

[12] Daniel L, Sichez H, Giorg R (2001). Tubular lesions and tubular cell adhesion molecules for the prognosis of lupus nephritis. *Kidney Int.*.

[13] Austin HA, Muenz LR, Joyce KM, Antonovych TT, Balow JE (1984). Diffuse proliferative lupus nephritis: identification of specific pathologic features affecting renal outcome. *Kidney Int.*.

[14] Ross S, Benz K, Sauerstein K, Amann K, Dötsch J, Dittrich K (2012). Unexpected recovery from long-term renal failure in severe diffuse proliferative lupus nephritis. *BMC Nephrol.*.

[15] Ayodele OE, Okpechi IG, Swanepoel CR (2013). Long-term renal outcome and complications in South Africans with proliferative lupus nephritis. *Int. Urol. Nephrol.*.

